# Supplementary material for: Utilization of Genetic Resources, Genetic Diversity and Genetic Variability for Selecting New Restorer Lines of Rice (Oryza sativa L.)
Source: Genes (Basel). 2022 Nov 27;13(12):2227. doi: 10.3390/genes13122227 (PMC9778048; doi:10.3390/genes13122227)
Supplement: Supplementary file 1 [file genes-13-02227-s001.zip › genes-1954718-supplementary.pdf]

Table S1. Pollen and spikelet fertility of hybrid combinations developed by crossing between IR79156A and G 46A cytoplasmic male sterile lines with new restorer lines.

Traits<br>crosses	IR79156A		G 46A	
	Pollen fertility (%)	Spikelet fertility (%)	Pollen fertility (%)	Spikelet fertility (%)
G1	91.5	88.96	91.4	88.49
G2	88.8	83.98	86.8	76.35
G3	89.23	78.5	90.4	80.45
G4	90.6	84.64	92.3	88.75
G5	95.53	93.07	91	84.94
G6	90.4	85.63	91.2	86.84
G7	95.8	93.13	88.12	78,7
G8	94.7	92.76	89.3	82.66
G9	95.1	93	86.7	78.46
G10	88.1	78.36	86.61	77.13
G11	97.4	96.06	95.77	90.58
G12	91.5	81.32	86.43	75.19
G13	87.66	78.11	86	77.21
G14	90.44	83.39	86.8	78.32
G15	86.51	77.24	91	85.21
G16	88.3	79.7	92.1	89.17
G17	88.14	79.22	87.6	78.75
G18	87.84	78.43	87.4	78.24

Table S2. Mean performance of days to 50% heading (day), plant height, number of panicles/plant of twenty rice genotypes in first, second years and combined across the two years

Genotypes	days to 50% heading			plant height (cm)			number of panicles/plant		
	First year	Second year	Combined	First year	Second year	Combined	First year	Second year	Combined
**G1**	105.0	105.5	105.3	97.13	91.00	94.07	15.2	14.4	14.8
**G2**	107.3	107.7	107.5	102.00	105.67	103.83	14.8	13.8	14.3
**G3**	130.0	130.5	130.2	100.43	105.00	102.72	15.6	15.0	15.3
**G4**	115.8	116.3	116.1	101.43	90.83	96.13	17.0	15.0	16.0
**G5**	116.5	117.1	116.8	105.57	98.83	102.20	18.8	16.3	17.6
**G6**	118.1	118.5	118.3	114.30	105.80	110.05	15.5	15.0	15.2
**G7**	102.8	103.4	103.1	102.60	99.33	100.97	15.0	13.2	14.1
**G8**	109.6	110.2	109.9	115.40	105.10	110.25	14.5	15.3	14.9
**G9**	109.0	109.6	109.3	152.20	138.27	145.23	17.9	15.0	16.5
**G10**	117.3	117.8	117.6	108.07	98.73	103.40	17.5	16.0	16.8
**G11**	108.7	109.2	108.9	112.80	102.77	107.78	17.4	15.5	16.5
**G12**	119.0	119.6	119.3	107.30	98.00	102.65	15.0	14.9	15.0
**G13**	101.0	101.5	101.2	111.27	96.33	103.80	16.2	14.1	15.2
**G14**	114.0	114.6	114.3	101.87	90.20	96.03	21.3	19.3	20.3
**G15**	118.5	119.0	118.7	115.87	98.47	107.17	15.8	17.1	17.7
**G16**	107.3	107.9	107.6	114.50	104.10	109.30	18.2	15.9	17.1
**G17**	117.8	118.3	118.1	131.43	122.07	126.75	20.8	18.6	19.7
**G18**	110.0	110.5	110.2	122.93	112.17	117.55	18.9	17.4	18.1
**G19**	79.4	79.9	79.7	89.53	88.14	88.84	12.6	12.0	12.3
**G 20**	104.3	104.8	104.6	111.47	109.80	110.63	15.6	14.4	15.0
**L.S.D. 5%**	1.3	1.0	1.1	2.90	3.98	2.81	1.3	1.3	1.2
**L.S.D. 1%**	1.8	1.3	1.5	3.88	5.33	3.77	1.8	1.7	1.6

L.S.D. : The least significant difference

Table S3. Mean performance of panicle length (cm), panicle weight (g), and pollen fertility (%) of twenty rice genotypes in first, second years and combined across the two years

Genotypes	panicle length (cm)			panicle weight (g)			pollen fertility (%)		
	First year	Second year	Combined	First year	Second year	Combined	First year	Second year	Combined
**G1**	23.4	23.3	23.4	4.3	4.1	4.2	93.75	97.79	95.77
**G2**	25.0	22.1	23.5	3.6	3.0	3.3	92.19	97.19	94.69
**G3**	25.0	26.8	25.9	4.5	5.0	4.8	92.72	93.59	93.16
**G4**	25.1	25.7	25.4	3.4	3.1	3.3	94.73	92.60	93.67
**G5**	25.5	25.8	25.6	3.1	3.0	3.0	93.21	91.42	92.32
**G6**	23.2	21.5	22.4	3.5	4.1	3.8	93.71	93.54	93.63
**G7**	23.5	22.1	22.8	4.8	4.1	4.4	85.78	88.70	87.24
**G8**	25.8	23.1	24.5	3.3	2.5	2.9	88.68	91.95	90.32
**G9**	25.1	25.8	25.5	5.8	6.2	6.0	94.60	91.81	93.21
**G10**	26.5	26.2	26.4	3.6	3.7	3.7	84.41	88.41	86.41
**G11**	27.2	25.4	26.3	5.3	4.2	4.8	92.07	83.92	88.00
**G12**	27.1	25.3	26.2	3.4	3.9	3.7	81.39	89.20	85.30
**G13**	27.4	25.5	26.5	4.8	5.0	4.9	81.91	89.15	85.53
**G14**	26.1	24.3	25.2	3.9	4.3	4.1	84.45	91.97	88.21
**G15**	25.3	24.2	24.7	3.7	3.3	3.5	89.67	89.65	89.66
**G16**	26.8	25.4	26.1	4.1	5.0	4.5	88.99	94.19	91.60
**G17**	26.8	25.1	26.0	3.6	4.2	3.9	91.76	95.22	93.49
**G18**	27.3	27.0	27.1	4.7	4.5	4.6	81.49	90.37	85.93
**G19**	21.3	22.6	22.0	3.4	3.3	3.4	85.88	87.09	86.49
**G 20**	24.8	25.4	25.1	3.8	3.5	3.7	86.72	87.84	87.28
**L.S.D. 5%**	1.3	1.5	1.1	0.7	0.5	0.5	2.96	2.80	2.01
**L.S.D. 1%**	1.8	2.0	1.5	0.9	0.7	0.7	3.97	3.76	2.70

L.S.D. : The least significant difference

Table S4. Mean performance of number of spikelets per panicle, spikelet fertility (%) and number of filled grains per panicle of twenty rice genotypes in first, second years and combined across the two years

Genotypes	**number of spikelets per panicle**			**spikelet fertility (%)**			**number of filled grains per panicle**		
	**First year**	**Second year**	**Combined**	**First year**	**Second year**	**Combined**	**First year**	**Second year**	**Combined**
G1	170.1	184.7	177.4	91.85	95.89	93.87	156.1	177.1	166.6
G2	142.5	135.9	139.2	90.29	95.29	92.79	128.6	129.5	129.1
G3	150.5	190.9	170.7	90.92	91.79	91.36	136.9	174.8	155.9
G4	141.7	152.2	147.0	92.73	90.60	91.67	131.4	138.0	134.7
G5	144.9	154.1	149.5	91.21	89.42	90.32	132.2	137.8	135.0
G6	145.0	152.6	148.8	91.61	91.44	91.53	132.7	139.4	136.0
G7	207.4	188.9	198.2	84.08	87.00	85.54	174.7	164.0	169.4
G8	185.6	166.7	176.2	87.18	90.45	88.82	161.5	150.8	156.2
G9	145.4	223.4	184.4	92.70	89.91	91.31	134.9	200.8	167.8
G10	141.5	119.1	130.3	82.45	86.45	84.45	116.4	103.0	109.7
G11	219.9	205.0	212.5	90.20	82.05	86.13	198.6	168.2	183.4
G12	168.9	142.2	155.6	79.29	87.10	83.20	133.8	123.8	128.8
G13	211.1	164.3	187.7	79.95	87.19	83.57	168.7	143.4	156.0
G14	145.7	128.3	137.0	82.60	90.12	86.36	120.2	115.6	117.9
G15	168.3	153.5	160.9	87.93	87.91	87.92	148.0	135.0	141.5
G16	167.5	141.6	154.5	86.79	91.99	89.40	145.4	130.2	137.8
G17	146.6	140.3	143.5	89.56	93.02	91.29	131.1	130.5	130.8
G18	161.7	160.3	161.0	79.39	88.27	83.83	128.3	141.5	134.9
G19	155.3	154.5	154.9	83.92	85.13	84.53	130.2	131.4	130.8
G 20	190.2	193.0	191.6	84.92	86.04	85.48	161.9	166.4	164.1
L.S.D. 5%	20.1	16.5	15.8	2.96	2.80	2.01	17.9	13.9	14.5
L.S.D. 1%	27.0	22.1	21.1	3.97	3.76	2.70	24.0	18.6	19.4

L.S.D. : The least significant difference

Table S5. Mean performance of 1000 grain weight (g) and grain yield per plant (g) of twenty rice genotypes in first, second years and combined across the two years

Genotypes	1000 grain weight (g)			grain yield per plant (g)		
	First year	Second year	Combined	First year	Second year	Combined
**G1**	23.00	22.17	22.59	35.65	38.15	36.90
**G2**	25.93	25.35	25.64	29.44	32.34	30.89
**G3**	25.24	24.94	25.09	30.60	33.60	32.10
**G4**	24.54	24.30	24.43	30.43	33.63	32.03
**G5**	25.33	24.92	25.13	34.18	37.58	35.88
**G6**	25.72	26.76	26.24	29.04	33.04	31.04
**G7**	25.73	25.56	25.65	38.19	41.09	39.64
**G8**	23.98	23.55	23.77	40.17	42.57	41.37
**G9**	29.12	28.94	29.03	32.32	35.42	33.87
**G10**	28.23	27.08	27.65	31.98	34.88	33.43
**G11**	24.75	24.82	24.79	49.42	51.62	50.52
**G12**	24.64	25.11	24.88	34.80	37.50	36.15
**G13**	23.71	24.13	23.92	52.92	55.42	54.17
**G14**	25.96	27.14	26.55	29.97	33.37	31.67
**G15**	25.20	25.28	25.24	42.33	45.13	43.73
**G16**	30.05	29.08	29.57	41.17	43.17	42.17
**G17**	27.58	28.19	27.89	46.85	49.65	48.25
**G18**	24.03	23.48	23.76	38.32	40.92	39.62
**G19**	23.66	23.52	23.59	30.67	33.77	32.22
**G 20**	21.00	21.04	21.02	38.72	41.12	39.92
**L.S.D. 5%**	1.50	1.00	0.93	4.42	4.42	4.42
**L.S.D. 1%**	2.01	1.34	1.24	5.92	5.92	5.92

L.S.D. : The least significant difference

Table S6. Mean performance of hulling percentage, milling percentage and amylose content percentage of twenty rice genotypes in first, second years and combined across the two years

Genotypes	Hulling percentage			Milling percentage			Amylase content percentage		
	First year	Second year	Combined	First year	Second year	Combined	First year	Second year	Combined
G1	80.22	81.12	80.67	68.27	69.17	68.72	16.07	16.97	16.52
G2	77.47	78.37	77.92	67.80	68.70	68.25	17.42	18.32	17.87
G3	79.20	80.20	79.70	70.40	71.40	70.90	24.31	25.31	24.81
G4	78.87	80.07	79.47	71.33	72.53	71.93	24.60	25.80	25.20
G5	77.20	78.30	77.75	69.07	70.17	69.62	25.08	26.18	25.63
G6	78.82	79.82	79.32	71.82	72.82	72.32	26.00	27.00	26.50
G7	81.60	82.30	81.95	69.73	70.43	70.08	23.70	24.40	24.05
G8	78.40	79.80	79.10	69.20	70.60	69.90	20.70	22.10	21.40
G9	79.80	80.41	80.11	72.80	73.41	73.11	28.66	29.27	28.97
G10	80.20	80.90	80.55	69.67	70.37	70.02	25.67	25.67	25.67
G11	78.87	80.07	79.47	68.13	69.33	68.73	19.03	20.23	19.63
G12	79.73	81.43	80.58	66.91	68.61	67.76	24.85	26.55	25.70
G13	78.53	80.03	79.28	69.67	71.17	70.42	17.29	18.79	18.04
G14	79.93	81.33	80.63	72.27	73.67	72.97	23.59	24.99	24.29
G15	80.87	81.15	81.01	70.60	70.88	70.74	24.00	24.28	24.14
G16	78.33	79.53	78.93	69.31	70.51	69.91	24.76	25.96	25.36
G17	78.80	79.60	79.20	71.67	72.47	72.07	28.14	28.94	28.54
G18	79.27	79.87	79.57	70.52	71.12	70.82	26.24	26.84	26.54
G19	79.29	80.39	79.84	68.49	69.59	69.04	25.97	27.07	26.52
G 20	79.63	80.63	80.13	68.54	69.54	69.04	28.68	29.68	29.18
L.S.D. 5%	1.20	1.20	1.20	1.10	1.10	1.10	1.12	1.12	1.12
L.S.D. 1%	1.61	1.61	1.61	1.47	1.47	1.47	1.50	1.50	1.50

L.S.D. : The least significant difference
